# Valorization of Beetroot Waste via Subcritical Water Extraction for Developing Active Food Packaging Materials

**DOI:** 10.3390/molecules30091928

**Published:** 2025-04-26

**Authors:** Márcia Correa de Carvalho, Pedro A. V. Freitas, Rosa J. Jagus, María V. Agüero, Amparo Chiralt

**Affiliations:** 1 Institute of Food Engineering Food UPV, Universitat Politècnica de València, 46022 Valencia, Spain; mcorrea.ext@fi.uba.ar (M.C.d.C.); pedvidef@doctor.upv.es (P.A.V.F.); 2 Consejo Nacional de Investigaciones Científica y Técnicas (CONICET), C.A.B.A., Buenos Aires C1428EGA, Argentina; mvaguero@fi.uba.ar; 3 Laboratorio de Investigación en Tecnología de Alimentos (C1428EGA), Instituto de Tecnologías y Ciencias de la Ingeniería (INTECIN), Facultad de Ingeniería, Departamento de Ingeniería Química, Universidad de Buenos Aires, C.A.B.A., Buenos Aires C1428EGA, Argentina; rjagus@fi.uba.ar

**Keywords:** active extracts, thermoplastic starch, poly (lactic acid), biodegradable packaging, oil oxidation

## Abstract

Obtaining active extracts from beet root leaves and stems (BLS) is an alternative for the valorization of this agricultural waste. Subcritical water extraction (SWE) at 150 °C and 170 °C has been used to obtain these extracts, which were incorporated (6% wt.) into polymer matrices to produce antioxidant films of thermoplastic starch (TPS) and polylactic acid (PLA) for the preservation of sunflower oil. A high extraction yield (67–60% solubilized solids) was achieved, and the extracts contained high levels of total phenols (51–73 mg GAE·g^−1^ extract) and betalains and great radical scavenging capacity (EC_50_: 30–22 mg mg^−1^ DPPH). The highest temperature promoted the extract’s phenolic richness and antioxidant capacity. The TPS and PLA films containing extracts exhibited color and UV-light blocking effects. The extracts reduced the oxygen permeability (OP) and water vapor permeability of PLA films while promoting those of the TPS films. The capacity of the films to preserve sunflower oil from oxidation was mainly controlled by the OP values of the films, which were very high in TPS films with low OP values. However, in the PLA films (which were more permeable to oxygen), the antioxidant extracts provided significant protection against sunflower oil oxidation.

## 1. Introduction

The valorization of by-products produced along the agri-food supply chain is a key approach in the circular economy, as the reuse of waste facilitates its conversion into valuable resources [1]. Likewise, the integral use of agri-food production is a sustainable approach to reduce losses. In particular, the waste generated from processing fruits and vegetables, including peels, seeds, leaves, and stems, has been utilized as soil fertilizer or animal feed and, more recently, as biomass for energy or fuel generation [2]. In addition to being highly inexpensive, these byproducts stand out for their high content of a wide range of bioactive compounds recognized for their potent antioxidant, antiradical, and antibacterial properties [3,4].

Beetroot plants are cultivated worldwide to obtain their roots, which generate large amounts of unexploited byproducts. Previous studies have shown that the beetroot leaves and stems (BLS), which constitute 50% of the whole plant, are usually discarded as waste [5]. However, these parts could be used as a source of bioactive compounds, mainly polyphenols and betalains [6], which could be recovered, thus improving the efficiency of plant utilization and increasing the sustainability of production systems and the agri-food chain. Maravić et al. [7] reported the bioactive characteristics of several phenolic acids from beet leaves, such as *p*-coumaric, sinapic, ferulic, vanillic, and syringic acids, as well as flavonoids, including vitexin, isovitexin, flavones, and catechin.

Different techniques can be applied to extract compounds from plants. Conventional methods, such as Soxhlet extraction, mechanical agitation, and maceration, utilize large amounts of organic solvents, requiring long extraction times and high operational handling [8]. Additionally, the use of organic solvents has been extensively questioned in recent years due to the environmental impact of their use and the safety aspects linked to their handling. Hence, there is a growing interest in developing more environmentally friendly, cleaner, safer, and easier extraction techniques [9]. In this context, emerging and innovative extraction technologies are widely used at the laboratory scale due to their ability to achieve higher extraction yields, shorter extraction times, and better preservation of the biological activity of polyphenols [7]. Novel technological processes applied for byproduct valorization are characterized by reduced energy consumption and environmental impact. Different processes, such as supercritical fluid extraction [10], high-pressure processing [11], pulsed electric fields [12], and ultrasound [13], have been suggested as interesting alternatives due to their ability to shorten the processing time, increase recovery yield, improve product quality, and enhance the functionality of extracts [14].

One of the most promising techniques for recovering bioactive compounds from byproducts on a large scale is subcritical water extraction (SWE) [15]. SWE is described as a highly efficient and selective extraction technique, where temperature and pressure are the most important factors for the success of the process [16]. Likewise, SWE is a cost-effective and environmentally friendly process that can be applied to agro-industrial waste, thus converting materials with no direct economic value into valuable resources. SWE has been widely used to extract polyphenolic compounds from various plant leaves, and its main limitations are the possible degradation of heat-sensitive compounds and the neo-formation of compounds due to heat-induced reactions [16]. Freitas et al. [17] obtained phenol-rich extracts from almond skin with high antioxidant capacity and antibacterial activity that could be used for different industrial applications.

The active extracts can be used in different fields, including as food additives or in active food packaging materials. In order to obtain more sustainable packaging materials that extend the shelf life of food, several studies have focused on the use of biodegradable polymers, such as thermoplastic starch (TPS) or polylactic acid (PLA), incorporating active extracts with antioxidant or antibacterial properties to obtain active packaging materials [18,19,20,21]. This strategy is also in line with current consumer demand for non-synthetic additives and contributes to proper waste management by producing value-added materials and reducing food losses [22].

In this sense, the aim of this study was to obtain active extracts from beetroot leaves and stems using SWE under different extraction conditions and to characterize them in terms of their extraction yield, total betalain and phenolic content, and antioxidant capacity. These extracts were also used to obtain active films based on two polymer matrices with different hydrophobicity, specifically PLA and TPS, in order to assess the effect of the extracts on relevant properties of the materials for food packaging applications, such as barrier capacity to oxygen and water vapor, light-blocking effect, and antioxidant activity. The latter was analyzed through their effectiveness in preserving packaged sunflower oil by applying an accelerated oxidation test.

## 2. Results and Discussion

### 2.1. Extraction Yields and Bioactive Properties of the Extracts

**Figure 1:**
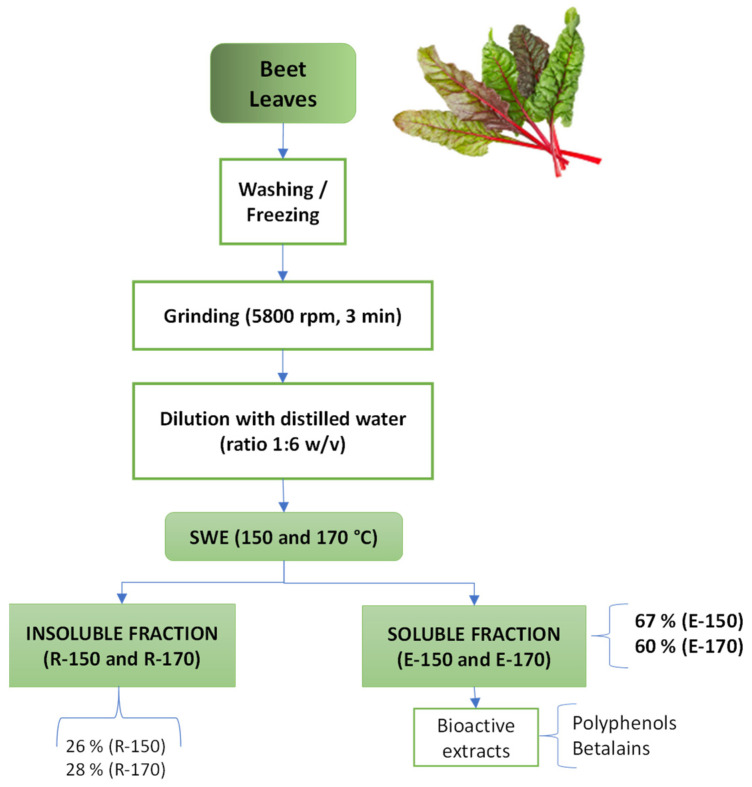
Flowchart of the process used for beetroot leaves and stems (BLS) fractionation, showing the yields (g outgoing solids; 100 g^−1^ of incoming dried material) of extracts (soluble fractions: E-150 and E-170) and residues (insoluble fractions: R-150 and R-170) obtained in SWE at 150 °C and 170 °C.

Figure 1 shows the flowchart of the process applied to the BLS to obtain active extracts using subcritical water under different extraction conditions, showing the yields of solids extracted and the extraction residue for each temperature (150 and 170 °C), referred to as the wt. percentage of the total BLS solids. Both extraction conditions promoted high solubilization of plant matrix components, with values of 67 ± 2 and 60 ± 2% solubilized solids at 150 °C and 170 °C, respectively. This significant (*p* < 0.05) difference in extract yield as the extraction temperature changed can be attributed to the differences in the extraction capacity of water, associated with changes in dielectric constant, surface tension, and viscosity [23]. Goyeneche et al. [24] reported similar levels of solid yield (~50%) by applying supercritical CO_2_ extraction (35 °C, 300 bar; and 50 °C, 400 bar) to an ethanolic dispersion of beet leaves, demonstrating the efficiency of the applied SWE method. Other authors [7] studied different techniques for extracting polyphenols from beetroot leaves, including liquid-solid extraction, ultrasound, microwave, pressurized liquid, and SWE, reporting lower solid yield values than those found in the present study.

The insoluble fraction after SWE was similar for both temperatures, representing 26 ± 3 and 28 ± 4% (for 150 °C and 170 °C, respectively) of the initial BLS solids. From the mass balance of the process, approximately 93–87% of the input material was recovered, indicating that some organic compounds could be partially degraded during the SWE step, generating volatiles such as acetic or formic acids from the hydrolysis of acetylated or methoxylated compounds [17]. Likewise, high SWE temperatures can promote the hydrolysis of peptide bonds in the proteins present in the plant matrix, leading to the release of amino acids that can participate, together with free sugars, in non-enzymatic browning reactions, such as Maillard reactions [25].

The total phenolic content (TPC), antioxidant activity (EC_50_ parameter), and betalain content of the different active BLS extracts are shown in Table 1. The TPC values, expressed as mg GAE per g of BLS extract solids, indicate the phenolic richness of each extract. In this context, the BLS extract obtained at 170 °C showed a higher TPC value (about 43% higher) than that obtained at 150 °C. This increase in the polyphenol content as the temperature rises aligns with findings obtained by other authors regarding SWE of different matrices [26,27]. Higher extraction temperatures, increase the diffusion coefficient of water within the plant matrix, as well as its ability to break chemical bonds between phenolic compounds and the lignocellulosic matrix, including ester, ether, and acetal covalent bonds [28]. Likewise, high temperatures increase the solubility of less polar compounds, such as phenolic compounds, thus increasing the TPC values [6,29]. Several studies have shown the presence of different phenolic compounds with bioactive properties in the leaves and stems of beetroot, making it an interesting source of phenols and justifying the exploitation and valorization of this agro-industrial waste. Zein et al. [30] identified several phenolic compounds with antioxidant and anticancer properties in aqueous extracts from beetroot leaves, such as vanillinic, pyrogallic, ellagic, protocatechuic, salicylic, benzoic, and chlorogenic acids. Other flavonoids were also reported, such as rosmarinic acid, vitexin, iuteolin, hesperidin, and nariginin.

**Figure 2:**
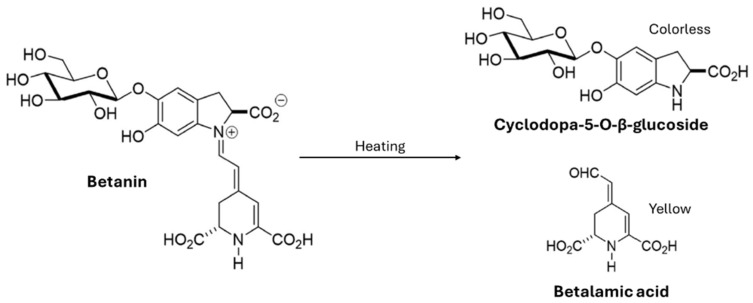
Betanin degradation through heating.

Regarding the betalain content, expressed as the betaxanthin (BX) and betacyanin (BC) concentrations, the highest temperature led to greater extraction of both components (*p* < 0.05). This can be attributed to the more extensive destruction of the BLS matrix, which leads to better exposure of the plant tissue and, therefore, greater extraction of betalains. In both SWE conditions, the BX concentration was higher than the BC content, which could be attributed to the greater thermostability of BX compared to BC, as observed by other authors [6,31]. Sanchez-Gonzalez et al. [32] studied the extraction of betalains from *Opuntia joconostle* cv. and reported that when betanin (betanidin 5-O-β-glucoside), a type of betacyanin, is heated above 60 °C for long periods, its hydrolysis in solution is accelerated, producing betalamic acid (yellow) and cyclodopa-5-O-β-glucoside (colorless) (Figure 2). Kumorkiewicz and Wybraniec [33] also reported that temperatures above 50 °C can thermodegrade the betacyanins through isomerization and/or decarboxylation, leading to a loss of color and antioxidant capacity.

The antioxidant capacity of the BLS extracts obtained by SWE was analyzed through the DPPH radical scavenging capacity and evaluated in terms of the EC_50_ parameter, which quantifies the amount of compound per mass unit of radical that inhibits the DPPH radical by 50%. As shown in Table 1, the higher the extraction temperature, the greater the radical scavenging capacity of the BLS extract. The EC_50_ value for E-170 was significantly lower (26%) than that of E-150, suggesting that less E-170 extract is required to reduce the DPPH radical concentration by 50%. Maravić et al. [7] also reported a marked increase (~300%) in the antioxidant capacity of beetroot leaf extracts when the temperature of the SWE process increased. The authors observed that the SWE temperature was the most important factor in obtaining extracts with radical scavenging capacity, as it affects both the plant matrix and the solvent properties. This tendency also agrees with that reported by Freitas et al. [17], who applied SWE to obtain active extracts from almond skin at 160 and 180 °C; the higher the extraction temperature, the lower the EC_50_ value of the extract.

Therefore, increasing the SWE extraction temperature significantly improved the extraction of phenolic compounds and betalains, as well as the antioxidant capacity of the extracts. It is worth mentioning that the extraction of non-phenolic components, including proteins, sugars, waxes, and ashes, can negatively affect the bioactive capacity of the obtained extracts due to the dilution effect of the phenolic compounds. Likewise, under high-temperature conditions, the present sugars and proteins can give rise to compounds derived from non-enzymatic browning reactions, typically caramelization and Maillard reactions. These neo-formed compounds, such as hydroxymethylfurfural, as well as the reaction intermediates, also exhibit bioactive properties, such as antioxidant and antimicrobial activities [34,35]; thus, they can contribute to the bioactive properties of the extracts.

In brief, SWE gave rise to BLS extracts with a high content of phenolic compounds that possess antioxidant properties. The extract obtained via SWE at 170 °C showed the highest levels of phenolics and betalains, as well as the greatest radical scavenging capacity. Therefore, these phenolic-rich extracts were used to create active and biodegradable food packaging materials, thus contributing to the valorization of waste and the production of plastic materials with a lower environmental impact.

### 2.2. Optical Properties of the Films Containing BLS Extracts

In order to investigate the effect of BLS extracts in different (hydrophobic and hydrophilic) polymer matrices, PLA and thermoplastic starch (TPS) films were prepared by incorporating 6% wt. of extract powder. Table 2 shows the visual appearance and color coordinates, specifically L*, C_ab_*, and h_ab_*, of PLA and TPS films with and without BLS extracts, as well as the total color difference (∆E_a_*) provoked by the extract, with respect to the respective extract-free films. Observation with the naked eye shows that both neat, colorless TPS and PLA films became brownish-yellow, darker, and more intense in color after the incorporation of the extract. This color change was more noticeable in the films with the E-170 extract, which exhibited a browner appearance. Therefore, the colored compounds in the extracts, mainly lignin and neo-formed brown compounds resulting from non-enzymatic browning reactions, provide the films with a colored appearance. The lignin fraction is responsible for the dark color of many plants [36]. The active films of both PLA and TPS matrices exhibited a significant reduction in luminosity (PLA films: 75.6 to 32–36; TPS films: 69 to 33–39), a marked increase in color saturation (PLA films: 4 to 19–25; starch films: 8 to 17–22), and changes in hue angle values (*p* < 0.05). The total color differences (∆E_ab_*) conferred by the extracts ranged from 33–37 for PLA films and 46 for TPS films. Other authors reported similar behavior for different films containing extracts obtained by SWE [18,20].

**Figure 3:**
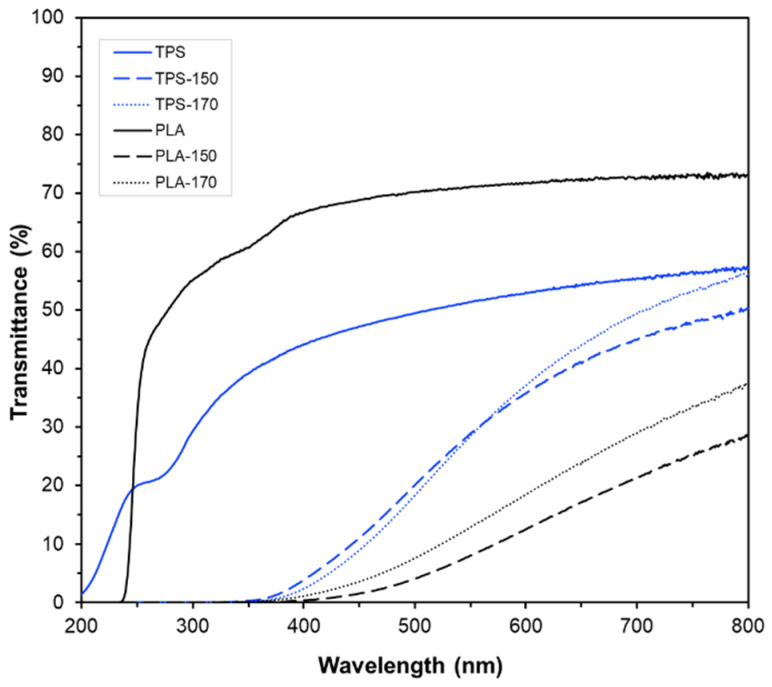
UV–vis transmittance spectra of PLA and TPS films, with and without BLS extracts (E-150 and E-170) at 6% wt., for the different films.

The transmission spectra in the UV–vis range (200–800 nm) of the films with and without BLS extracts are shown in Figure 3. In both matrices, the transparent, extract-free PLA and TPS films exhibited no selective light absorption for wavelengths above 250 nm, with the PLA film being more transparent than the starch one. Other authors have also reported this spectral behavior, which can be attributed to a homogeneous material that does not have dispersed particles or colored compounds within the polymer matrix [37]. Nonetheless, incorporating BLS extracts significantly affected the light transmittance of the films. The active films showed intense absorbance in the visible wavelength region, while a total blocking effect of ultraviolet radiation up to 350 nm was observed. Specifically, the active TPS films showed lower light absorption in the visible region than the PLA films, despite their lower inherent transparency. This suggests a different integration/interaction of the extract compounds in the polymer matrix that affects light interactions. In particular, the hydrophilic nature of the starch matrix may contribute to better compatibility with the extract’s polar compounds, thus producing more homogeneous blends with lower light scattering effects. In contrast, the more hydrophobic nature of PLA makes homogeneous blending more difficult, promoting the presence of dispersed extract particles in the matrix, which produce light scattering, decrease light transmission, and enhance film opacity. Freitas et al. [19] observed the presence of micro-particles in PLA films containing rice straw extract.

The light-blocking effect of the active films is attributable to both the presence of colored compounds in the extracts, which absorb in the visible region, and phenolic compounds that absorb UV radiation. In fact, the conjugated double bonds and aromatic rings present in the chemical structure of phenolic compounds are responsible for this UV-blocking behavior [38]. Thus, the incorporation of extracts into both polymers provides the films with an additional benefit, as they can prevent UV-induced oxidation of packaged foods, such as the oxidation of vitamins, pigments, and fatty acids, thereby offering a promising solution for food preservation.

### 2.3. Barrier Properties of the Films Containing BLS Extracts

Table 3 shows the oxygen permeability (OP) and water vapor permeability (WVP) values of PLA and TPS films with and without BLS extracts. In general, PLA films have a high barrier to water vapor but not to oxygen, whereas starch films are highly permeable to water vapor but exhibit a good barrier to oxygen. The incorporation of BLS extract promoted significant (*p* < 0.05) changes in the barrier properties of the films, which were dependent on the polymer matrix. For the films based on PLA, the extracts reduced the OP and WVP values, while in TPS films, the extracts slightly increased their permeabilities. This suggests a different interaction between the components of the extracts and the polymer chains. The possible interactions between the phenolic compounds and PLA chains have been described through hydrogen bonding of the phenolic OH with the polyester carbonyls, which may lead to chain crosslinking [21,39]. Thus, the interactions between phenolic compounds and the polymer promoted a decrease in molecular mobility and, therefore, the diffusion phenomena responsible for mass transport in the permeation process. This behavior can be observed in the WVP values of PLA films with E-150 and E-170 extracts, which showed a significant (*p* < 0.05) reduction of 45 and 34%, respectively. The hydrophilic nature of the extract components, as well as the oxygen scavenging capacity of the phenolic compounds [40], also could have limited the oxygen solubility and its mass transfer rate through the PLA matrix. This effect was more noticeable for the PLA-150 film, which showed a significant (*p* < 0.05) reduction in the OP value of 14%, compared to the extract-free PLA film. Although the E-150 extract did not have the highest phenolic content, other more hydrophilic components, such as sugars, may have affected the solubility of oxygen molecules within the PLA matrix.

In contrast, the hydrophilic compounds of the extracts compatible with the starch matrix could lead to a plasticizing effect on the starch chain network, thus increasing molecular mobility and the mass transport rate through the polymer matrix. Likewise, the increase in the mass transport rate can also be due to a partial hydrolysis of the starch chains during thermoprocessing, promoted by the presence of phenolic acids, as observed in previous studies of starch films containing sunflower hull extracts [41]. The partial hydrolysis of the polymer chains would reduce the cohesive forces of the polymer network, thus increasing permeability. This behavior may explain the decline in the barrier capacity of the TPS films with E-150 and E-170 extracts, which showed a significant (*p* < 0.05) increase in OP of 40 and 32%, respectively, and a significant (*p* < 0.05) increase in WVP values of 42 and 62%, respectively.

Therefore, the incorporation of BLS extracts caused marked changes in the WVP and OP of the films. The PLA films with extract exhibited higher oxygen and water vapor barrier capacity, while the TPS films with extract showed an increase in both permeabilities. These results could affect the stability of packaged foods, either accelerating or slowing down the deterioration process during storage.

### 2.4. Antioxidant Capacity of the Films Containing BLS Extracts

**Figure 4:**
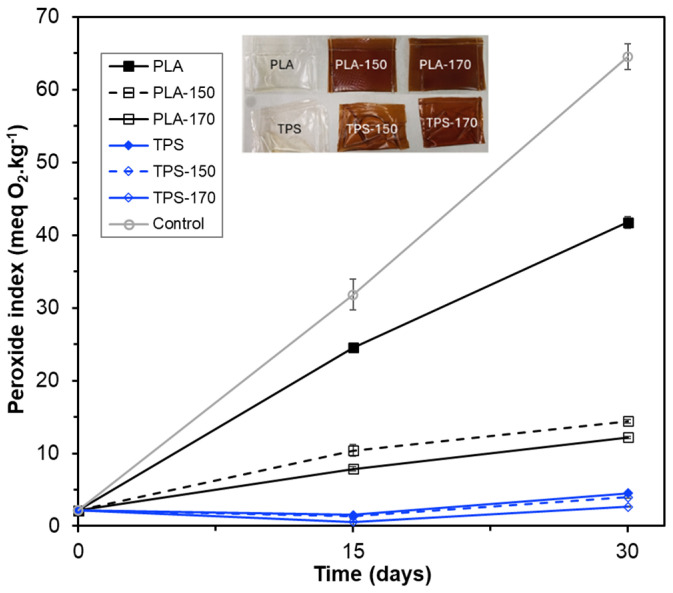
Visual appearance of the different thermo-sealed bags and the peroxide index (PI) values of sunflower oil packaged in PLA and TPS bags, with and without BLS extract, obtained via SWE at 150 and 170 °C at 6% wt.

The antioxidant capacity of active films was evaluated on real food through their ability to prevent sunflower oil oxidation when subjected to an accelerated oxidation test. To this end, packaged sunflower oil samples in vacuum heat-sealed single-dose bags were exposed to accelerated oxidation conditions for 30 days. Extract-free films based on PLA or starch, as well as open Petri dishes containing sunflower oil samples, were used as controls. Figure 4 shows the visual appearance of the active and control bags and the progress of the peroxide index (PI) of the packaged and unpackaged sunflower oil samples over 30 days. PI is a good indicator of oil quality, as it quantifies hydroperoxides formed during the first stages of oxidation of unsaturated fatty acids [42]. A marked and progressive increase in PI values was observed for the unpackaged oil samples, thus validating the highly oxidizing conditions of the test.

The PI values of all the packaged samples were kept below those observed for the open control samples, showing the preservative effect of the films, with or without active extracts. The starch-based films, whether containing extract or not, were the most effective in slowing down the generation of peroxides in the packaged samples. This result is attributable to the higher oxygen barrier capacity of TPS films compared to PLA films. In fact, the lower permeability of oxygen molecules through the polymer matrix was the most important factor in preserving oil samples packaged with TPS films. The active starch films, especially the TPS-170, promoted a slight decrease in peroxide values of the samples compared to the control TPS film. This may be due to the combined effect of both the release of antioxidant phenolic compounds into the oil samples and the UV light-blocking effect of the films. The PI values of the samples packed with the starch films remained below 10 meq O_2_·kg^−1^ sample throughout the test, which indicates that they were stable against oxidation [42]. Other authors [20] also observed a good capacity of starch-based bags for the preservation of sunflower oil oxidation.

PLA films with higher oxygen permeability exhibited lower preservation capacity for sunflower oil oxidation. In contrast, the antioxidant effect of the incorporated extracts was more noticeable. Compared with the extract-free PLA film, the PLA films with extracts were markedly effective in controlling the levels of peroxides in the sunflower oil samples. This preservation effect was greater in the samples packaged with the PLA-170 film than in those in contact with the PLA-150 film, despite the non-significant differences in the OP values of both materials and their similar UV light-blocking effects. However, the antioxidant capacity of the extract E-170 was higher than that of E-150 (Table 1). Therefore, this result points to the action of released antioxidant compounds from the PLA-170 film, which has a higher antioxidant character, into the oil samples, inhibiting the free radicals formed during the oil oxidation process to a greater extent. It is worth mentioning that the peroxide levels of the samples in contact with the PLA-170 film reached 10 meq·kg^−1^ after about 30 days of storage, whereas those packaged with the PLA-150 film showed values above this limit after 15 days of storage.

Therefore, the incorporation of BLS extracts into films based on different biodegradable polymer matrices, such as PLA and TPS, represents a promising alternative for producing antioxidant and biodegradable food packaging materials. This approach considers aspects related to reducing environmental impact, valorizing agro-industrial waste, and promoting a circular economy.

## 3. Materials Methods

### 3.1. Chemicals

Corn starch (~27% amylose) was supplied by Roquette (Roquette Laisa, Benifaió, Spain). Amorphous PLA 4060D, with a density of 1.24 g·cm^−3^ and an average molecular weight of 106,226 D, was purchased from Natureworks (Blair, NE, USA). Glycerol, acetic acid, diphosphorus pentoxide (P_2_O_5_), and magnesium nitrate (Mg(NO_3_)_2_) were supplied by PanReac Quimica S.L.U. (Castellar del Vallés, Spain). Gallic acid, Folin–Ciocalteau reagent (2 N), methanol (>99.9% purity), 2,2-diphenyl-1-picrylhydrazyl (DPPH), sodium chlorite, 2-thiobarbituric acid (>98% purity), and 1,1,3,3-tetramethoxypropane were purchased from Sigma-Aldrich (St. Louis, MO, USA). Iodine (99.5% purity) was obtained from Acros Organics^®^ (Geel, Belgium). McIlvaine buffer was prepared using disodium phosphate and citric acid.

### 3.2. Plant Preparation

Beetroot leaves and stems (*Beta vulgaris* L. var. conditiva) (BLS) were obtained from Alboraya (Valencia, Spain) beet fields. The leaves and stems were washed with water and frozen (at −18 ± 2 °C) until use. Before extraction, the partially frozen plant material was milled using a Thermomix TM-5 (Vorwerk Spain M.S.L., S.C., Madrid, Spain) at 5800 rpm for 3 min. Likewise, the initial water content was determined by drying the samples at 80 °C until a constant weight was achieved, which was found to be 91.3 wt% (wet basis). This water content was used to determine the solids–water ratio in the reactor after the incorporation of distilled water.

### 3.3. Subcritical Water Extraction

SWE was applied in duplicate to the BLS under two extraction conditions: 150 °C, 4.8 bar (E-150), and 170 °C, 9 bar (E-170), both at 150 rpm for 30 min (Figure 1). These temperatures were selected on the basis of previous studies that analyzed the optimal temperature range for phenol extraction in plant matrices by SWE [16]. For the extraction, the fresh crushed BLS were mixed with distilled water at a ratio of 1:6 (*w*/*v*) to enhance the agitation of the mixture and mass transfer, and placed in a pressure reactor (Model 1-T-A-P-CE, 5 L capacity, Amar Equipment PVT.LTD, Mumbai, India). The actual solid-water ratio in the reactor was 1:79.4, considering the water contributed by the leaves. Afterwards, each plant dispersion was vacuum filtered using a qualitative filter (Filterlab S.A., Catalonia, Spain), and the soluble fractions were freeze-dried (Telstar, model LyoQuest-55, Barcelona, Spain) at −60 °C, 0.8 mbar for 72 h to obtain the powdered BLS extracts. The obtained powders were stored in a dark bottle at 4 ± 2 °C until further use.

### 3.4. Extract Characterization

The BLS extracts were characterized by their total solid yield (TSY), total phenolic content (TPC), betalain content, and antioxidant capacity. The TSY was obtained in duplicate by drying an aliquot of each BLS soluble fraction after SWE at 60 °C under vacuum for 12 h (until constant weight). The total extracted solids were obtained from the determined solid/water ratio multiplied by the total water content in the reactor (which includes the BLS water content plus the added water). The results were expressed as g dry extract·100 g^−1^ BLS solids.

The total phenolic content (TPC) of the BLS extracts was determined using the Folin–Ciocalteu method, as previously described [21]. The results were expressed as mg of gallic acid equivalent (GAE) per 100 g of BLS extract solids.

The antioxidant capacity of the extracts was evaluated using the 2,2-diphenyl-1-pikryl-hydrazyl (DPPH) radical scavenging method [43] and expressed as the EC_50_ value (mg BLS extract·mg^−1^ DPPH). The EC_50_ parameter is defined as the ratio of the sample to DPPH required to reduce the initial concentration of DPPH by 50% when reaction stability is achieved.

The betalain content of the BLS extracts was obtained spectrophotometrically following a previously described method [13]. For this analysis, the extracts were diluted in McIlvaine buffer, and the absorbance was measured at wavelengths of 476, 536, and 600 nm. The betaxanthin (BX) and betacyanin (BC) contents were expressed as µg vulgaxanthin-I per g of BLS extract and µg betanin per g of BLS extract, respectively. This analysis was performed in triplicate for each BLS extract.

### 3.5. Film Preparation

#### 3.5.1. Thermoplastic Starch (TPS) Films

Thermoplastic starch-based films (about 150 μm thick) were obtained using glycerol as a plasticizer at 30% wt., relative to the total starch mass. The proportion of the extracts in the films (6% wt. relative to the polymer) was established on the basis of previous studies [18,19,20,21], where other plant extracts with similar antioxidant capacity, when incorporated into polymeric matrices, provided the films with antioxidant properties with minor changes in other functional properties of the material, such as barrier or mechanical performance. The films were obtained by melt blending the components followed by compression molding, as described by Freitas et al. [18], with some modifications. Pre-conditioned starch at 53% relative humidity (RH) and 25 °C for 7 days was first mixed with the other film components and melt-blended in an internal mixer (HAAKETM PolyLabTM QC, Thermo Fisher Scientific, Karlsruhe, Germany) at 130 °C and 50 rpm for 10 min. For each formulation, the obtained solid mixture was cold ground (using liquid nitrogen) in a Thermomix TM-5 (Vorwerk Spain M.S.L., S.C., Madrid, Spain) and conditioned at 25 °C and 53% RH for one week. After that, the ground powder (4 g per film) was placed onto Teflon sheets and compression-molded in a heat plate hydraulic press (Model LP20, Labtech Engineering, Samutprakam, Thailand) as follows: preheating at 160 °C for 1 min, compression at 50 bar and 160 °C for 2 min, followed by 110 bar for 4 min, and then a final cooling for 3 min to about 70 °C. All films were conditioned at 25 °C and 53% RH before characterization. As a control material, neat starch films (without extracts) were prepared. Therefore, three starch formulations were obtained: starch without extract (TPS), starch with E-150 (TPS-150), and starch with E-170 (TPS-170).

#### 3.5.2. Amorphous PLA Films

Films based on PLA (about 150 μm thick) were also obtained via melt blending and compression molding, as reported by Freitas et al. [18,19], with some modifications. PLA pellets were dried in a vacuum oven (vacuum TEM-TJP Selecta, Barcelona, Spain) at 60 °C for 12 h to eliminate residual water. Then, the PLA pellets were melt-blended with BLS extracts (E-150 and E-170) at 6% wt. relative to the total polymer mass in an internal mixer (HAAKETM PolyLab TM QC, Thermo Fisher Scientific, Herzogenaurach, Germany) at 160 °C, with a rotation speed of 50 rpm for 6 min. The obtained solid mass was cold ground in a Thermomix TM-5 (Vorwerk Spain M.S.L., S.C., Madrid, Spain), and 3 g of each formulation were placed onto Teflon sheets and compression-molded in a heat plate hydraulic press (Model LP20, Labtech Engineering, Thailand) as follows: preheating at 160 °C for 3.5 min, compression at 100 bar and 160 °C for 3 min, followed by final cooling for 3 min to about 70 °C. All films were conditioned at 25 °C and 53% RH until use. PLA films without extracts were obtained as control films. Thus, three film formulations were obtained: PLA without extract (PLA), PLA with E-150 (PLA-150), and PLA with E-170 (PLA-170).

### 3.6. Characterization of the Film Properties

#### 3.6.1. Barrier Properties

The OP of the films was determined in duplicate according to the ASTM D3985-05 methodology [44] using an Oxygen Permeation Analyzer (Model 8101e, Systech Illinois, Lisle, IL, USA) at 25 °C and 53% RH. The measured area of the films was 50 cm^2^, and the oxygen transmission rate (*OTR*) was recorded every 15 min until equilibrium was reached. The *OP* of the films was determined according to Equation (1), which considers the thickness of the film sample and the difference in partial pressure of oxygen (∆*p*) between the two sides of the film.

(1) $$OP=\frac{OTR}{∆p}\times thickness$$

The water vapor permeability (WVP) of the films was obtained gravimetrically, in triplicate, according to ASTM E96/E96M [45]. Conditioned film samples (53% RH and 25 °C for one week) were cut (Ø = 3.5 cm) and sealed in Payne permeability cups (Elcometer SPRL, Hermelle/s Argenteau, Belgium) containing 7 mL of distilled water (100% RH) and placed into desiccators at 25 °C and 53% RH (using over-saturated Mg(NO_3_)_2_). The cups were weighed periodically (ME36S, Sartorius, ±0.00001 g, Fisher Scientific, Hampton, NH, USA) every 1.5 h until 27 and 48 h for the starch and PLA films, respectively. The WVP of the films was determined from the water vapor transmission rate (WVTR), which was calculated from the slope of the weight loss vs. time curve and corrected, as described by Mchugh et al. [46] for the effect of concentration gradients established in the stagnant air gap inside the cup. The water vapor permeance of each film sample was calculated according to Equation (2) as a function of the vapor pressure gradient between the inner (p_2_) and outer (p_3_) surfaces of the film in the desiccator. The WVP of the films was obtained by multiplying the permeance by the average film thickness.

(2) $$Permeance=\frac{WVTR}{\left(p_{2}-p_{3}\right)}$$

#### 3.6.2. Optical Properties

The optical properties of the films were obtained based on the Kubelka–Munk theory of multiple scattering using a spectrocolorimeter (CM-3600d, Minolta Co., Osaka, Japan). The reflection spectra of the films, ranging from 400 to 700 nm, were obtained on white (R_g_) and black (R_0_) backgrounds, and the infinite reflectance spectra (R∞) were obtained according to Equations (3)–(6). The film color coordinates L* (lightness), a* (redness–greenness), and b* (yellowness–blueness) were obtained from the R_∞_ spectra, considering an observer at 10° and illuminant D65. Finally, the psychometric color coordinates, chroma (C_ab_*) (Equation (7)), hue angle (h_ab_*) (Equation (8)), and total color difference (ΔE*) of each film, relative to the respective control film without extract, were calculated (Equation (9)).

(3) $$T_{i}=\sqrt{{\left(a-R_{0}\right)}^{2}-b^{2}}$$

(4) $$R_{\infty }=a-b$$

(5) $$a=\frac{1}{2}\;\left[R+\left(\frac{R_{0}-R+R_{g}}{R_{0}\times R_{g}}\right)\right]\;$$

(6) $$b=\sqrt{a^{2}-1}$$

(7) $$C^{*}=\sqrt{a^{*2}+b^{*2}}$$

(8) $$h^{*}=arctg\left(\frac{b^{*}}{a^{*}}\right)$$

(9) $$∆E^{*}=\sqrt{\left({\left(∆L^{*}\right)}^{2}+{\left(∆a^{*}\right)}^{2}+{\left(∆b^{*}\right)}^{2}\right)}$$

Here, ΔL* = (L* − L*_0_); Δa* = (a* − a*_0_); Δb* = (b* − b*_0_); and L*_0_, a*_0_, and b*_0_ are the color coordinates of the starch or PLA films without extracts.

The light barrier properties of the films were evaluated through UV–vis spectra ranging from 200 to 800 nm, using a UV–visible spectrophotometer (Evolution 201, Thermo Scientific, Waltham, MA, USA) operating in light transmission mode.

#### 3.6.3. Antioxidant Properties of the Film

The antioxidant capacity of the active films was analyzed through their capacity to prevent the oxidation of packaged sunflower oil under accelerated oxidation conditions [20]. To this end, films of TPS and PLA, with or without BLS extracts, were used to obtain mono-dose oil bags (7 cm × 11 cm) using a vacuum heat-sealer (Vacio Press, Saeco, Molina de Segura, Spain). Subsequently, 7 mL of commercial sunflower oil was placed into the bags and heat-sealed (Figure 4). The different bags were stored in a chamber at 30 °C and 53% RH and exposed to fluorescent light (intensity of 1000–1500 lx) at a distance of 30 cm from the samples for 30 days. The peroxide index of the packaged samples was evaluated on different storage days (0, 15, and 30) in triplicate for each type of bag and time. The oxidative conditions were confirmed in control samples consisting of open Petri dishes containing 7 mL of sunflower oil.

The peroxide index (PI) of sunflower oil samples was quantified in duplicate using the titration method (IUPAC—International Union of Pure & Applied Chemistry). For this approach, a 1 g oil sample was dissolved in 10 mL of glacial acetic acid–decan-1-ol at a ratio of 3:2 (*v*/*v*), and saturated potassium iodide solution (200 μL) was added and kept in the dark for 1 min. Afterward, 50 mL of distilled water was added, and the dispersion was titrated using an automatic titrator (Titrando, Metrohm Ion Analysis, Herisau, Switzerland) with sodium thiosulfate (0.01 M or 0.001 M, depending on the expected peroxide concentration).

### 3.7. Statistical Analysis

The experimental data were submitted to analysis of variance (ANOVA) using the Minitab Statistical Program (version 17), considering a 95% confidence level. Tukey’s studentized range (HSD) test, with a significant difference (α) of at least 5%, was applied.

## 4. Conclusions

The results highlighted the value of recovering plant byproducts, such as beetroot leaves and stems, by means of new extraction technologies that are environmentally friendly and free of toxic solvents, such as SWE. Increasing the SWE temperature from 150 to 170 °C favored the polyphenol extraction yields, the betalain content, and the antioxidant capacity of the extracts. The application of SWE proves to be a suitable strategy for intensifying the extraction of bioactive compounds from BLS.

The incorporation of extracts into the PLA and starch matrices provides the films with color and a total UV light-blocking effect, which is beneficial for preserving food against oxidation reactions. When incorporated into the PLA matrix, the extracts reduced oxygen and water vapor permeabilities, while slightly worsening the barrier properties of the starch films. The capacity of the films to preserve sunflower oil from oxidation was mainly affected by the oxygen permeability of the films, which is almost 30 times higher for the PLA matrices. Therefore, oxidation was very limited in samples packaged in starch-based films with very low oxygen permeability. However, in the PLA films, the ability to block UV light, combined with the action of antioxidant compounds from the polymer matrix, allows for the enhancement of their antioxidant capacity, preserving sunflower oil from oxidation. Of the PLA formulations, the one containing the extract obtained at 170 °C (with the highest radical scavenging capacity) was the most effective in slowing down oxidation reactions. Therefore, the development of biodegradable active materials for the packaging of oxidation-sensitive products constitutes a viable alternative for valorizing beetroot waste. Further studies with oxidation-prone foods and different barrier requirements are needed to validate the materials obtained in terms of their ability to extend product shelf life.

## Figures and Tables

**Table 1:** Total phenolic content (TPC), betaxanthin (BX), and betacyanin (BC) contents and DPPH scavenging capacity (EC_50_) of the BLS extracts (E-150 and E-170) obtained via subcritical water extraction at 150 °C and 170 °C.

Extract	TPC (mg GAE·g^−1^ Extract)	EC_50_ (mg Extract·mg^−1^ DPPH)	Betalains	
			BX (µg·g^−1^ Extract)	BC (µg·g^−1^ Extract)
E-150	51.3 ± 1.8 ^b^	30.1 ± 0.2 ^a^	752 ± 16 ^b^	396 ± 15 ^b^
E-170	73.2 ± 1.9 ^a^	22.2 ± 0.2 ^b^	900 ± 30 ^a^	456 ± 17 ^a^

Different letters in the same column indicate significant differences between films according to the Tukey test (α = 0.05).

**Table 2:** Colour coordinates (lightness: L*, chrome: C_ab_*, and hue angle: h_ab_*) and total global differences (ΔE_ab_*) in PLA and starch (TPS) films with and without BLS extracts obtained at 150 °C and 170 °C.

Formulation	Visual Appearance	L*	C_ab_*	h_ab_*	∆E*
PLA	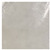	75.6 ± 0.1 ^a^	4.2 ± 0.1 ^c^	15.3 ± 0.6 ^b^	-
PLA-150	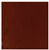	32.0 ± 0.4 ^b^	19.2 ± 0.3 ^b^	58.2 ± 0.6 ^a^	33.2 ± 0.4 ^b^
PLA-170	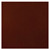	36.0 ± 6.9 ^b^	25.4 ± 9.1 ^a^	60.8 ± 2.7 ^a^	37.2 ± 0.4 ^a^
TPS	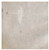	69.7 ± 1.4 ^a^	8.2 ± 0.1 ^c^	60.1 ± 1.2 ^b^	-
TPS-150	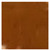	39.7 ± 0.6 ^b^	22.4 ± 0.4 ^a^	63.9 ± 0.3 ^a^	46.5 ± 0.3 ^a^
TPS-170	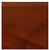	33.6 ± 0.4 ^c^	17.1 ± 0.3 ^b^	55.0 ± 0.3 ^c^	46.3 ± 1.4 ^a^

Different letters in the same column indicate significant differences between films according to the Tukey test (α = 0.05).

**Table 3:** Oxygen (OP) and water vapor (WVP) permeabilities of PLA and TPS films with and without BLS extracts (E-150 and E-170) at 6% wt.

Formulation	OP ×10^14^ (cm^3^·m^−1^·s^−1^·Pa^−1^)	WVP ×10^11^ (g·Pa^−1^·s^−1^·m^−1^)
PLA	188.0 ± 2.0 ^a^	10.0 ± 2.0 ^a^
PLA-150	162.3 ± 1.2 ^c^	5.5 ± 1.2 ^b^
PLA-170	182.0 ± 3.0 ^b^	6.6 ± 2.0 ^b^
TPS	6.5 ± 0.1 ^b^	248 ± 40 ^b^
TPS-150	9.1 ± 1.6 ^a^	351 ± 40 ^a^
TPS-170	8.6 ± 0.1 ^a^	401 ± 13 ^a^

Different letters in the same column indicate significant differences between films according to the Tukey test (α = 0.05).

## Data Availability

The original contributions presented in the study are included in the article. Further inquiries can be directed to the corresponding author.
